# Supplementary material for: Modeling glioblastoma heterogeneity as a dynamic network of cell states
Source: Mol Syst Biol. 2021 Sep 16;17(9):e10105. doi: 10.15252/msb.202010105 (PMC8444284; doi:10.15252/msb.202010105)
Supplement: Supplementary file 5 — Source Data for Figure 3 [file MSB-17-e10105-s001.zip › Figure3A_sourcedata/GSEA_3065/nosigdb_state1.GseaPreranked.1622718409535/GAROFANO_IAVARONE_NEU_SUBTYPE.html]

Details for gene set GAROFANO\_IAVARONE\_NEU\_SUBTYPE[GSEA]

|  || Dataset | state1 |
| Phenotype | NoPhenotypeAvailable |
| Upregulated in class | na\_neg |
| GeneSet | GAROFANO\_IAVARONE\_NEU\_SUBTYPE |
| Enrichment Score (ES) | -0.68671894 |
| Normalized Enrichment Score (NES) | -2.5712466 |
| Nominal p-value | 0.0 |
| FDR q-value | 0.0 |
| FWER p-Value | 0.0 |
Table: GSEA Results Summary

  

Fig 1: Enrichment plot: GAROFANO\_IAVARONE\_NEU\_SUBTYPE      
 Profile of the Running ES Score & Positions of GeneSet Members on the Rank Ordered List

  

| PROBE | GENE SYMBOL | GENE\_TITLE | RANK IN GENE LIST | RANK METRIC SCORE | RUNNING ES | CORE ENRICHMENT || 1 | EPHB2 |  |  | 1148 | 0.118 | -0.1130 | No |
| 2 | KIF26B |  |  | 1444 | 0.093 | -0.1393 | No |
| 3 | PFN2 |  |  | 1462 | 0.091 | -0.1370 | No |
| 4 | SMAP2 |  |  | 1586 | 0.083 | -0.1460 | No |
| 5 | CA10 |  |  | 1674 | 0.077 | -0.1516 | No |
| 6 | CACNG4 |  |  | 2647 | 0.035 | -0.2502 | No |
| 7 | ZMIZ1 |  |  | 2887 | 0.028 | -0.2736 | No |
| 8 | SH3RF3 |  |  | 3033 | 0.024 | -0.2874 | No |
| 9 | HMX1 |  |  | 3458 | 0.015 | -0.3304 | No |
| 10 | EEPD1 |  |  | 3518 | 0.013 | -0.3359 | No |
| 11 | KDM1B |  |  | 3558 | 0.013 | -0.3394 | No |
| 12 | PRKAB2 |  |  | 3625 | 0.011 | -0.3457 | No |
| 13 | FHOD3 |  |  | 3786 | 0.008 | -0.3618 | No |
| 14 | CDK5R1 |  |  | 3866 | 0.007 | -0.3696 | No |
| 15 | WASF1 |  |  | 3937 | 0.006 | -0.3766 | No |
| 16 | CDO1 |  |  | 4212 | 0.001 | -0.4048 | No |
| 17 | NUDT11 |  |  | 4390 | -0.003 | -0.4229 | No |
| 18 | RAB9B |  |  | 4430 | -0.003 | -0.4268 | No |
| 19 | GADD45G |  |  | 4503 | -0.005 | -0.4340 | No |
| 20 | JARID2 |  |  | 4539 | -0.005 | -0.4374 | No |
| 21 | BMP7 |  |  | 4562 | -0.006 | -0.4394 | No |
| 22 | DAB1 |  |  | 4601 | -0.006 | -0.4430 | No |
| 23 | MIIP |  |  | 4618 | -0.006 | -0.4444 | No |
| 24 | SSH2 |  |  | 4696 | -0.007 | -0.4520 | No |
| 25 | CCDC88C |  |  | 4734 | -0.008 | -0.4555 | No |
| 26 | NCOA2 |  |  | 4751 | -0.008 | -0.4567 | No |
| 27 | FAM107B |  |  | 4798 | -0.009 | -0.4611 | No |
| 28 | DCC |  |  | 4919 | -0.011 | -0.4729 | No |
| 29 | TBC1D12 |  |  | 4924 | -0.011 | -0.4729 | No |
| 30 | CSMD2 |  |  | 4978 | -0.012 | -0.4778 | No |
| 31 | TMEM170B |  |  | 5055 | -0.013 | -0.4850 | No |
| 32 | MAPRE2 |  |  | 5063 | -0.013 | -0.4851 | No |
| 33 | RPAIN |  |  | 5176 | -0.015 | -0.4960 | No |
| 34 | MNX1 |  |  | 5303 | -0.017 | -0.5082 | No |
| 35 | CCDC28B |  |  | 5523 | -0.020 | -0.5299 | No |
| 36 | RASAL2 |  |  | 5684 | -0.023 | -0.5453 | No |
| 37 | KIF2A |  |  | 5712 | -0.024 | -0.5471 | No |
| 38 | TSTD2 |  |  | 5962 | -0.029 | -0.5714 | No |
| 39 | SATB1 |  |  | 6070 | -0.030 | -0.5811 | No |
| 40 | PARP8 |  |  | 6079 | -0.030 | -0.5806 | No |
| 41 | GNG2 |  |  | 6136 | -0.032 | -0.5850 | No |
| 42 | MBTD1 |  |  | 6198 | -0.033 | -0.5898 | No |
| 43 | RAP2A |  |  | 6372 | -0.036 | -0.6061 | No |
| 44 | MED13L |  |  | 6507 | -0.039 | -0.6182 | No |
| 45 | FBXL16 |  |  | 6544 | -0.039 | -0.6201 | No |
| 46 | PJA1 |  |  | 6636 | -0.041 | -0.6277 | No |
| 47 | YLPM1 |  |  | 6760 | -0.044 | -0.6384 | No |
| 48 | ANKRD13B |  |  | 6776 | -0.045 | -0.6380 | No |
| 49 | ATG2B |  |  | 6822 | -0.045 | -0.6406 | No |
| 50 | CELF2 |  |  | 6823 | -0.045 | -0.6386 | No |
| 51 | MARCKSL1 |  |  | 6861 | -0.046 | -0.6404 | No |
| 52 | CPT1C |  |  | 6924 | -0.048 | -0.6447 | No |
| 53 | FOXN3 |  |  | 7031 | -0.051 | -0.6533 | No |
| 54 | AKT3 |  |  | 7063 | -0.052 | -0.6542 | No |
| 55 | FAM110B |  |  | 7201 | -0.055 | -0.6659 | No |
| 56 | PTPRD |  |  | 7218 | -0.056 | -0.6651 | No |
| 57 | EZH1 |  |  | 7314 | -0.058 | -0.6723 | No |
| 58 | GSK3B |  |  | 7351 | -0.059 | -0.6734 | No |
| 59 | ZNF532 |  |  | 7388 | -0.060 | -0.6745 | No |
| 60 | PTBP2 |  |  | 7463 | -0.062 | -0.6793 | No |
| 61 | LMBR1L |  |  | 7464 | -0.062 | -0.6766 | No |
| 62 | USP24 |  |  | 7529 | -0.064 | -0.6804 | No |
| 63 | MAPK8IP3 |  |  | 7546 | -0.065 | -0.6792 | No |
| 64 | TMOD2 |  |  | 7576 | -0.066 | -0.6793 | No |
| 65 | RBPJ |  |  | 7588 | -0.066 | -0.6775 | No |
| 66 | RALGPS2 |  |  | 7602 | -0.066 | -0.6759 | No |
| 67 | GRIA4 |  |  | 7708 | -0.070 | -0.6836 | Yes |
| 68 | ARVCF |  |  | 7714 | -0.070 | -0.6811 | Yes |
| 69 | BRWD3 |  |  | 7718 | -0.071 | -0.6783 | Yes |
| 70 | C5orf30 |  |  | 7755 | -0.072 | -0.6788 | Yes |
| 71 | RNF144A |  |  | 7775 | -0.072 | -0.6776 | Yes |
| 72 | PEG10 |  |  | 7803 | -0.073 | -0.6771 | Yes |
| 73 | C20orf194 |  |  | 7810 | -0.074 | -0.6745 | Yes |
| 74 | DNER |  |  | 7852 | -0.075 | -0.6754 | Yes |
| 75 | KIAA0895L |  |  | 7861 | -0.076 | -0.6729 | Yes |
| 76 | TNIK |  |  | 7901 | -0.077 | -0.6735 | Yes |
| 77 | CLN8 |  |  | 7909 | -0.078 | -0.6708 | Yes |
| 78 | NXPH1 |  |  | 7920 | -0.078 | -0.6684 | Yes |
| 79 | SEC61A2 |  |  | 7931 | -0.079 | -0.6660 | Yes |
| 80 | ZNF248 |  |  | 7957 | -0.080 | -0.6650 | Yes |
| 81 | PDE10A |  |  | 7959 | -0.080 | -0.6616 | Yes |
| 82 | HMBOX1 |  |  | 7964 | -0.080 | -0.6585 | Yes |
| 83 | ZSWIM6 |  |  | 8018 | -0.082 | -0.6604 | Yes |
| 84 | KIF3A |  |  | 8087 | -0.084 | -0.6636 | Yes |
| 85 | GSTA4 |  |  | 8147 | -0.087 | -0.6659 | Yes |
| 86 | HIP1R |  |  | 8149 | -0.087 | -0.6622 | Yes |
| 87 | ENHO |  |  | 8176 | -0.089 | -0.6609 | Yes |
| 88 | PPP1R12B |  |  | 8180 | -0.089 | -0.6573 | Yes |
| 89 | ARHGAP33 |  |  | 8204 | -0.089 | -0.6558 | Yes |
| 90 | GRK4 |  |  | 8239 | -0.091 | -0.6552 | Yes |
| 91 | SBK1 |  |  | 8244 | -0.092 | -0.6516 | Yes |
| 92 | GABBR1 |  |  | 8312 | -0.095 | -0.6543 | Yes |
| 93 | RAB2A |  |  | 8354 | -0.097 | -0.6543 | Yes |
| 94 | WWC1 |  |  | 8400 | -0.099 | -0.6546 | Yes |
| 95 | GPT2 |  |  | 8405 | -0.099 | -0.6506 | Yes |
| 96 | SPAST |  |  | 8429 | -0.101 | -0.6486 | Yes |
| 97 | VPS8 |  |  | 8456 | -0.102 | -0.6467 | Yes |
| 98 | MTMR9 |  |  | 8480 | -0.104 | -0.6445 | Yes |
| 99 | CASK |  |  | 8532 | -0.107 | -0.6450 | Yes |
| 100 | KLHL24 |  |  | 8561 | -0.109 | -0.6431 | Yes |
| 101 | UBL3 |  |  | 8573 | -0.110 | -0.6394 | Yes |
| 102 | ACVR2B |  |  | 8601 | -0.112 | -0.6373 | Yes |
| 103 | CLIP3 |  |  | 8688 | -0.116 | -0.6410 | Yes |
| 104 | DAAM1 |  |  | 8691 | -0.117 | -0.6361 | Yes |
| 105 | DNM3 |  |  | 8701 | -0.117 | -0.6319 | Yes |
| 106 | SGSM2 |  |  | 8709 | -0.118 | -0.6274 | Yes |
| 107 | FHIT |  |  | 8710 | -0.118 | -0.6222 | Yes |
| 108 | MAPT |  |  | 8749 | -0.121 | -0.6207 | Yes |
| 109 | ABCC5 |  |  | 8787 | -0.124 | -0.6191 | Yes |
| 110 | CRMP1 |  |  | 8801 | -0.125 | -0.6150 | Yes |
| 111 | POU2F1 |  |  | 8841 | -0.128 | -0.6133 | Yes |
| 112 | CPEB4 |  |  | 8849 | -0.128 | -0.6084 | Yes |
| 113 | C2orf72 |  |  | 8855 | -0.129 | -0.6032 | Yes |
| 114 | ZNF300 |  |  | 8866 | -0.130 | -0.5985 | Yes |
| 115 | KIF1A |  |  | 8881 | -0.132 | -0.5942 | Yes |
| 116 | PHYHIPL |  |  | 8937 | -0.137 | -0.5938 | Yes |
| 117 | FAM19A5 |  |  | 8956 | -0.139 | -0.5895 | Yes |
| 118 | SENP7 |  |  | 8969 | -0.140 | -0.5846 | Yes |
| 119 | SHC3 |  |  | 8972 | -0.141 | -0.5786 | Yes |
| 120 | MMP15 |  |  | 9003 | -0.143 | -0.5754 | Yes |
| 121 | NIN |  |  | 9011 | -0.143 | -0.5698 | Yes |
| 122 | NLGN2 |  |  | 9039 | -0.146 | -0.5662 | Yes |
| 123 | TMSB15A |  |  | 9079 | -0.150 | -0.5636 | Yes |
| 124 | EVL |  |  | 9083 | -0.151 | -0.5572 | Yes |
| 125 | NF1 |  |  | 9090 | -0.151 | -0.5512 | Yes |
| 126 | CADM2 |  |  | 9141 | -0.157 | -0.5494 | Yes |
| 127 | DCLK1 |  |  | 9143 | -0.157 | -0.5426 | Yes |
| 128 | ZMYM2 |  |  | 9171 | -0.162 | -0.5383 | Yes |
| 129 | FAM171A2 |  |  | 9172 | -0.162 | -0.5312 | Yes |
| 130 | ATCAY |  |  | 9276 | -0.176 | -0.5340 | Yes |
| 131 | SPPL3 |  |  | 9311 | -0.183 | -0.5295 | Yes |
| 132 | PAG1 |  |  | 9322 | -0.185 | -0.5224 | Yes |
| 133 | PLXNB1 |  |  | 9326 | -0.186 | -0.5145 | Yes |
| 134 | ATF7IP |  |  | 9369 | -0.194 | -0.5103 | Yes |
| 135 | ARHGEF9 |  |  | 9378 | -0.196 | -0.5025 | Yes |
| 136 | CLASP2 |  |  | 9383 | -0.197 | -0.4942 | Yes |
| 137 | RERE |  |  | 9401 | -0.200 | -0.4871 | Yes |
| 138 | REV3L |  |  | 9431 | -0.208 | -0.4810 | Yes |
| 139 | NAV1 |  |  | 9460 | -0.216 | -0.4743 | Yes |
| 140 | PHLPP1 |  |  | 9463 | -0.217 | -0.4650 | Yes |
| 141 | ZNF462 |  |  | 9480 | -0.222 | -0.4569 | Yes |
| 142 | NAV2 |  |  | 9505 | -0.227 | -0.4494 | Yes |
| 143 | GPC2 |  |  | 9511 | -0.228 | -0.4399 | Yes |
| 144 | GRIN2B |  |  | 9584 | -0.254 | -0.4361 | Yes |
| 145 | KIF5C |  |  | 9597 | -0.257 | -0.4260 | Yes |
| 146 | DPP6 |  |  | 9606 | -0.262 | -0.4153 | Yes |
| 147 | TSHZ1 |  |  | 9633 | -0.273 | -0.4059 | Yes |
| 148 | ABAT |  |  | 9646 | -0.278 | -0.3950 | Yes |
| 149 | HIPK2 |  |  | 9660 | -0.286 | -0.3837 | Yes |
| 150 | KIDINS220 |  |  | 9662 | -0.289 | -0.3711 | Yes |
| 151 | PODXL2 |  |  | 9686 | -0.301 | -0.3602 | Yes |
| 152 | SRGAP3 |  |  | 9689 | -0.304 | -0.3470 | Yes |
| 153 | PCSK1N |  |  | 9695 | -0.309 | -0.3339 | Yes |
| 154 | SOX4 |  |  | 9704 | -0.318 | -0.3207 | Yes |
| 155 | SLC35F1 |  |  | 9715 | -0.327 | -0.3073 | Yes |
| 156 | DCLK2 |  |  | 9717 | -0.328 | -0.2930 | Yes |
| 157 | MTSS1 |  |  | 9741 | -0.350 | -0.2799 | Yes |
| 158 | ZEB2 |  |  | 9745 | -0.353 | -0.2647 | Yes |
| 159 | ITM2C |  |  | 9758 | -0.363 | -0.2500 | Yes |
| 160 | RUFY3 |  |  | 9765 | -0.372 | -0.2342 | Yes |
| 161 | NKTR |  |  | 9767 | -0.376 | -0.2178 | Yes |
| 162 | CHD7 |  |  | 9769 | -0.379 | -0.2012 | Yes |
| 163 | RTN3 |  |  | 9792 | -0.413 | -0.1853 | Yes |
| 164 | LSAMP |  |  | 9814 | -0.481 | -0.1663 | Yes |
| 165 | WSB1 |  |  | 9836 | -0.554 | -0.1440 | Yes |
| 166 | GRIA2 |  |  | 9846 | -0.638 | -0.1169 | Yes |
| 167 | NCAM1 |  |  | 9862 | -0.751 | -0.0853 | Yes |
| 168 | SOX6 |  |  | 9863 | -0.798 | -0.0502 | Yes |
| 169 | BCAN |  |  | 9878 | -1.175 | 0.0001 | Yes |
Table: GSEA details [plain text format]

  

Fig 2: GAROFANO\_IAVARONE\_NEU\_SUBTYPE: Random ES distribution      
 Gene set null distribution of ES for **GAROFANO\_IAVARONE\_NEU\_SUBTYPE**
